# The *Drosophila* homologue of CTIP1 (Bcl11a) and CTIP2 (Bcl11b) regulates neural stem cell temporal patterning

**DOI:** 10.1242/dev.200677

**Published:** 2022-09-07

**Authors:** Paul M. Fox, Jocelyn L. Y. Tang, Andrea H. Brand

**Affiliations:** The Gurdon Institute and Department of Physiology, Development and Neuroscience, University of Cambridge, Tennis Court Road, Cambridge CB2 1QN, UK

**Keywords:** Temporal patterning, Neural development, Neural stem cells, Chronophage, CTIP1/2, Bcl11a, Bcl11b

## Abstract

In the developing nervous system, neural stem cells (NSCs) use temporal patterning to generate a wide variety of different neuronal subtypes. In *Drosophila*, the temporal transcription factors, Hunchback, Kruppel, Pdm and Castor, are sequentially expressed by NSCs to regulate temporal identity during neurogenesis. Here, we identify a new temporal transcription factor that regulates the transition from the Pdm to Castor temporal windows. This factor, which we call Chronophage (or ‘time-eater’), is homologous to mammalian CTIP1 (Bcl11a) and CTIP2 (Bcl11b). We show that Chronophage binds upstream of the *castor* gene and regulates its expression. Consistent with Chronophage promoting a temporal switch, *chronophage* mutants generate an excess of Pdm-specified neurons and are delayed in generating neurons associated with the Castor temporal window. In addition to promoting the Pdm to Castor transition, Chronophage also represses the production of neurons generated during the earlier Hunchback and Kruppel temporal windows. Genetic interactions with Hunchback and Kruppel indicate that Chronophage regulates NSC competence to generate Hunchback- and Kruppel-specified neurons. Taken together, our results suggest that Chronophage has a conserved role in temporal patterning and neuronal subtype specification.

## Abstract (precis)

**Summary:** The *Drosophila* CTIP1/2 (Bcl11a/b) homologue *chronophage* regulates the temporal cascade and neural stem cell competence in the embryonic central nervous system.

## INTRODUCTION

During central nervous system development, individual neural stem cells (NSCs) give rise to distinct subtypes of neurons. Temporal patterning of NSCs regulates the sequential birth order of neuronal subtypes and, as a result, neuronal subtypes are produced in a defined order (Okano and Temple, 2009; Kohwi and Doe, 2013). Temporal patterning takes place in the mammalian cerebral cortex, where progenitors in the ventricular and subventricular zones sequentially give rise to the neurons that populate layers II-VI (Molyneaux et al., 2007; Leone et al., 2008; Greig et al., 2013). Neurons within the same layer share cell identities and a number of studies have identified layer-specific factors that regulate neuronal cell fate (Leyva-Díaz and López-Bendito, 2013). These include FEZF2 (Chen et al., 2005a,b), SOX5 (Lai et al., 2008) and CTIP2 (Arlotta et al., 2005), which regulate early-born neurons (deep cortical layers), and BRN1 and BRN2 (Sugitani et al., 2002), which regulate late-born neurons (superficial layers). Factors that act within the progenitor cells to determine temporal identity include the transcription factors Ikaros (Alsio et al., 2013) and FEZF2 (Chen et al., 2008), which have been shown to regulate the generation of relatively early born cortical neurons, and the chromatin remodelling factor SATB2 (Alcamo et al., 2008), which promotes later-born neurons. These factors are likely central players in the temporal regulation of cortical development, and their identification suggests that progression of temporal identity involves transcription factor cascades. However, the overall mechanism by which precursors acquire distinct temporal identities remains unclear.

Temporal identity has been extensively studied in the *Drosophila* embryonic ventral nerve cord (VNC) (for reviews, see Maurange, 2012; Kohwi and Doe, 2013; Doe, 2017; Miyares and Lee, 2019). NSCs within the VNC integrate both spatial and temporal signalling cues to generate defined lineages of neurons. Spatial signalling pathways assign a unique positional identity to each of the 30 NSCs within a hemisegment (Doe, 1992). As NSCs divide, they give rise sequentially to distinct subtypes of neurons (Bossing et al., 1996; Schmidt et al., 1997; Schmid et al., 1999). The sequential patterning of the lineages requires the temporal cascade transcription factors Hunchback (Hb), Krüppel (Kr), Nubbin and Pdm2 (collectively referred to as Pdm), and Castor (Cas) (Isshiki et al., 2001; Grosskortenhaus et al., 2006; Tran and Doe, 2008; Baumgardt et al., 2009). The expression of each factor corresponds to a unique temporal window, during which a lineage generates specific neuronal subtypes (Brody and Odenwald, 2000; Isshiki et al., 2001; Pearson and Doe, 2003).

Taking advantage of the ability to track individual neurons, previous studies have shown that the loss of a given temporal factor causes the neural stem cell to skip the generation of the corresponding subtypes (Isshiki et al., 2001; Novotny et al., 2002; Grosskortenhaus et al., 2006). One model lineage is NB7-1, in which the first five neural stem cell divisions give rise to the U motor neurons (U1-U5) in the corresponding numerical order. Hb specifies U1 and U2, Kr specifies U3, Pdm specifies U4 and Cas specifies U5. These neurons express Even-skipped (Eve) and can be identified based on spatial location and additional markers, thereby providing a functional readout of temporal specification. As an example, when Hb is lost, NB7-1 skips the generation of the U1 and U2 neurons, indicating that Hb specifies the fates of U1 and U2.

Genetic analysis of temporal identity in the VNC has uncovered two classes of factors, the aforementioned ‘temporal factors’, which are necessary for specification of their corresponding progeny, and ‘switching factors’, which promote the switch from one temporal window to the next. This second class of factors includes the transcription factors Svp, Dan and Danr, which promote the Hb to Kr transition (Kanai et al., 2005; Kohwi et al., 2011), and Pdm and Cas, which appear to have lineage specific roles as switching factors (Tran and Doe, 2008). Loss of these switching factors delays the transition from one temporal window to the next, thus prolonging the initial temporal window and leading to reiteration of its corresponding neuronal subtypes. For example, in the NB7-1 lineage, loss of Svp, Dan or Danr delays the Hb to Kr switch, and leads to increased numbers of Hb-specified U1/U2 neurons (Kanai et al., 2005; Kohwi et al., 2011).

Svp, Dan and Danr comprise two parallel pathways that regulate the switch from Hb to Kr (Kohwi et al., 2011), and the characterisation of Svp has revealed a mechanism by which NSCs coordinate cell division with temporal cascade progression (Mettler et al., 2006). Transcription of *svp* mRNA begins during the Hb window; however, its nuclear export is inhibited, and translation of *svp* mRNA requires mitosis (Mettler et al., 2006). The identification and characterisation of additional switching factors will be essential for a complete understanding of temporal cascade progression.

In this study we show that the gene *CG9650*, encoding the *Drosophila* homologue of the zinc-finger transcription factors CTIP1 (*Bcl11a*) and CTIP2 (*Bcl11b*) (Avram et al., 2000), acts as a switching factor in the embryonic VNC. We have named this factor Chronophage (Cph), for its role in temporal cascade progression. Cph promotes the transition from the Pdm to the Cas temporal window. Loss of *cph* (an alternative symbol for *CG9650*) delays the switch from Pdm to Cas and, in the NB7-1 lineage, this leads to the generation of excess Pdm-specified U4 motor neurons. Our data suggest that Cph promotes the switch by directly regulating Cas expression: neural stem cell expression of Cph begins immediately before Cas, Cph is necessary for Cas induction and Cph directly binds the *cas* locus. In addition to its role as a switching factor, Cph regulates neural stem cell competence to generate early born Hb- and Kr-specified neurons. Intriguingly, the homologue of Cph, CTIP2, is activated downstream of the temporal factor FEZF2 in the mammalian cerebral cortex (Chen et al., 2008). We propose that this family of genes has a conserved role in temporal patterning and neuronal subtype specification.

## RESULTS

### Cph is homologous to the zinc-finger transcription factors CTIP1 (Bcl11a) and CTIP2 (Bcl11b), and displays temporal-specific expression in the developing CNS

**Fig. 1.**
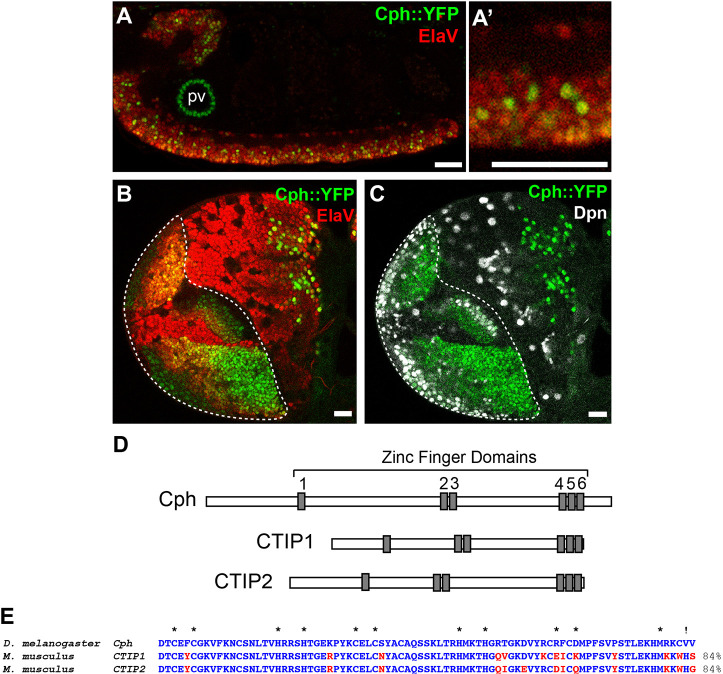
**Cph is the *Drosophila* homologue of CTIP1 and CTIP2.** (A,A′) Cph::YFP is expressed in a subset of neurons throughout the central brain and ventral nerve cord. Lateral views of a stage 16 embryo containing a protein trap insertion in *cph* (*cph::YFP*). Neurons express Elav (red). Expression is also detected in the proventriculus (pv). Scale bars: 20 μm. (B,C) Cph::YFP is expressed in the optic lobe NSCs and neurons (at wandering third instar larval stage). NSCs express Deadpan (Dpn in white). Cph::YFP expression was detected using an anti-GFP antibody. Scale bars: 20 μm. (D) Domain structure of Cph, CTIP1 and CTIP2 showing the arrangement of the six homologous zinc-finger domains (grey). (E) Amino acid alignment of the protein sequence spanning zinc fingers 4-6. Asterisks indicate the cysteine and histidine residues contained within the zinc-finger domains. Zinc finger 6 in Cph has a C_2_HC (!) rather than C_2_H_2_ sequence. C_2_H_2_-type zinc-finger proteins are the most common transcription factors in eukaryotes.

We screened a collection of protein trap lines for expression within the developing central nervous system and identified the line CPTI001740, which contains an insertion in the gene *CG9650*. Expression of *CG9650* was visible during both embryonic and larval central nervous system development and appeared to localise to a subset of neurons in the VNC and central brain (Fig. 1A,A′). In the larval optic lobe, *CG9650* expression was evident in neurons and NSCs in both the medulla and lamina (Fig. 1B,C). BLAST searches revealed that *CG9650* is homologous to the vertebrate genes CTIP1 (*Bcl11a*) and CTIP2 (*Bcl11b*), which encode zinc-finger transcription factors that contribute to development of the CNS (Avram et al., 2000; Arlotta et al., 2005; John et al., 2012). CTIP1 and CTIP2 share six homologous C_2_H_2_ zinc-finger domains. The central pair of zinc fingers (ZF3 and ZF4 in CTIP2) has been shown to confer DNA-binding specificity (Avram et al., 2002). These six zinc-finger domains are highly conserved in *CG9650* (Fig. 1D,E). *CG9650* is the single *Drosophila* orthologue of CTIP1 (*Bcl11a*) and CTIP2 (*Bcl11b*). The conserved DNA-binding zinc-finger domains and expression pattern suggest that *CG9650* has a conserved role in CNS development.

**Fig. 2.**
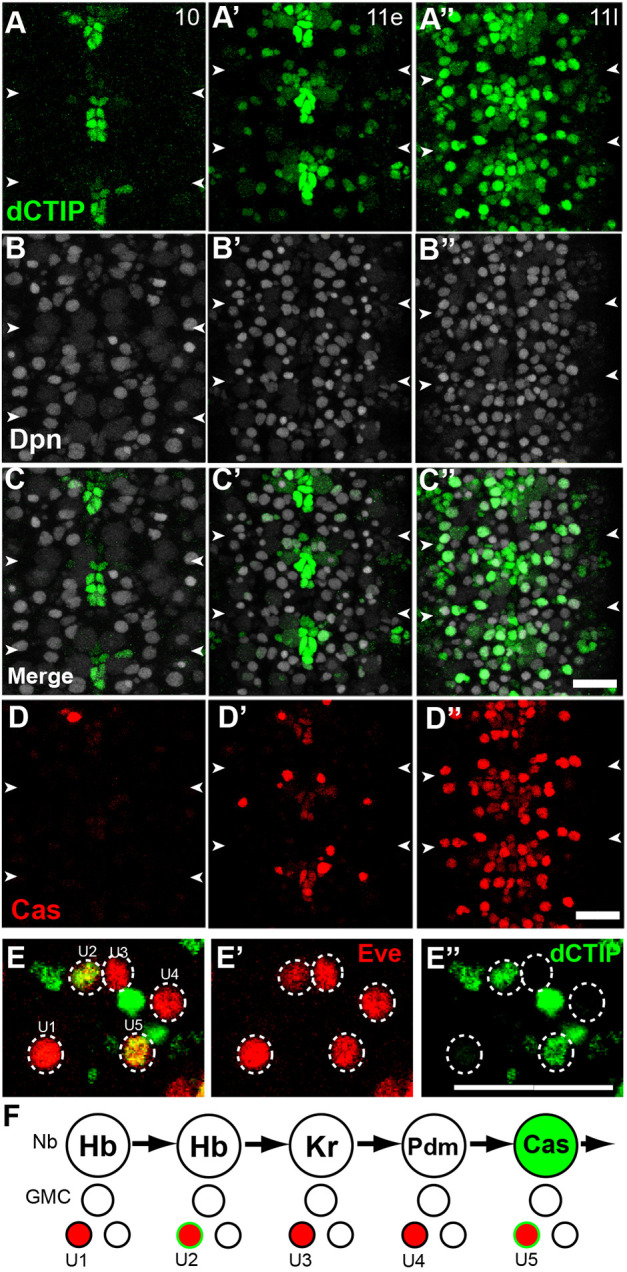
***cph* is expressed in a temporal pattern in VNC neural stem cells.** (A-D″) Ventral view of NSCs from segments T3-A2 at stages 10 (A-D), early 11 (A′-D′) and late 11 (A″-D″) stained for Cph::YFP (green), Cas (red) and Dpn (white, to indicate NSCs). For each stage, all channels are taken from the same embryo. *cph* expression begins at early/mid stage 11, and nearly all NSCs express Cph::YFP by late stage 11. At early stage 11 (A′-D′), when Cph::YFP can first be observed in a subset of NSCs, Cas is largely absent. White arrowheads indicate segmental boundaries. (E-E″) U neurons were identified by Eve expression (red) and spatial arrangement within the hemisegment. *cph* is expressed by U2 and U5. (F) Diagram of early NB7-1 lineage during U neuron generation. NB7-1 divides asymmetrically to produce a GMC. The GMC then divides to generate a U neuron and an Eve-negative sibling. The NSC sequentially expresses the temporal cascade factors Hb, Kr, Pdm and Cas, which specify the corresponding U neurons. Green indicates *cph* expression in NSCs and U neurons. Scale bars: 20 μm. *n*=6 embryos from three independent experiments.

Our initial observations of the GFP protein trap line suggested that *CG9650* is expressed in a temporal pattern within the NSCs of the VNC, and hereafter refer to it as *chronophage* (*cph*). We compared the timing of Cph expression with that of the previously characterised temporal cascade factors Hb, Kr, Pdm and Cas, which are expressed sequentially in NSCs during embryonic stages 8-12 (Brody and Odenwald, 2000; Isshiki et al., 2001). We found that NSCs begin to express Cph::YFP at stage 11, with expression continuing throughout embryogenesis (Fig. 2A-A″,C-C″). At stage 11, NSCs transition from expression of Pdm to Cas. Specifically, Cas expression in NSCs begins largely at late stage 11 (Fig. 2D-D″), and there is a brief period in which NSCs express both Pdm and Cas. Cph expression begins immediately before Cas expression, as demonstrated by the fact that we could observe a subset of NSCs at early stage 11 expressing Cph::YFP but not Cas (Fig. 2A′-C′). Therefore, the onset of Cph expression within the NSCs at stage 11 correlates with the transition from the Pdm temporal window to the Cas temporal window.

How does the expression of Cph in post-mitotic neurons correspond with its neural stem cell expression pattern? During each temporal window, NSCs generate neuronal subtypes that typically inherit the expression of the corresponding temporal factor. In the NB7-1 lineage, each U neuron with the exception of U4 inherits the expression of the corresponding temporal factor. We observed Cph::YFP expression in two distinct U neurons. Consistent with the observation that Cph expression coincides with Cas, Cph was expressed in U5 neurons (Fig. 2D,E). However, Cph was also expressed in U2 (Fig. 2D,E), which is specified during the earlier Hb temporal window (stages 8-9). As Cph is not expressed in NB7-1 at this stage, we conclude that Cph expression in neurons can either be inherited from the neural stem cell (as in the case of U5) or arise post-mitotically (as in the case of U2). Overall, the Cph expression pattern suggests that it plays a role in neural stem cell temporal identity.

### Cph is a temporal cascade switching factor that regulates the Pdm to Cas transition

**Fig. 3.**
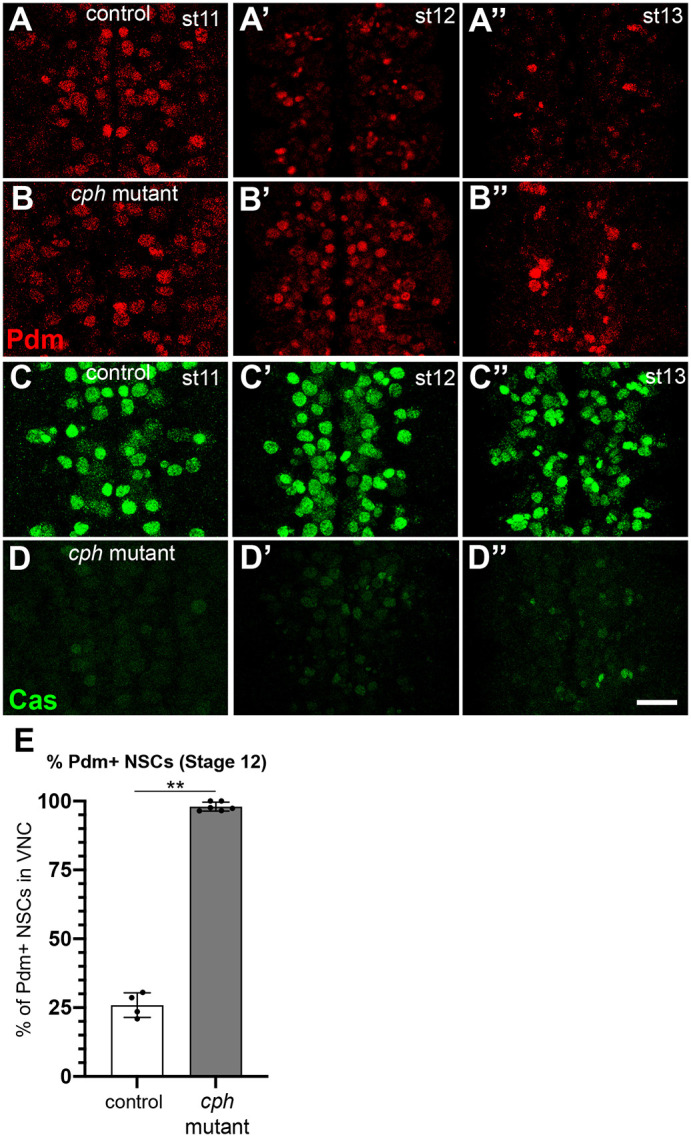
**Cph regulates the transition from Pdm to Castor.** Wild-type embryos transition from Pdm (red) to Cas (green) during stage 11. Loss of *cph* extends Pdm expression and decreases Cas expression. (A-A″) Control embryos express Pdm at stage 11 (A), stage 12 (A′) and partially at stage 13 (A″). (B-B″) In *cph* mutants, Pdm expression is elevated at stage 12 (B′) and continues until stage 13 (B″). (C-C″) Control embryos express Cas from stage 11 to stage 13. (D-D″) In *cph* mutants, Cas expression is strongly diminished. Ventral views of the NSC layer of control embryos (A-A″,C-C″) or *cph* mutants (B-B″,D-D″) stained for Pdm (red, A-B″) or Cas (green, C-D″) at stages 11, 12 and 13. Scale bar: 20 μm. (E) Quantification of the percentage of Pdm^+^ NSCs in the ventral side of the ventral nerve cord at stage 12 between control and *cph*. A Mann–Whitney test was used to test for statistical significance (**P<0.01). *n*=6 embryos from three independent experiments, except for A′ (*n*=4 embryos).

To determine whether loss of *cph* affected neural stem cell temporal identity, we created a *cph* deletion mutant via site-specific recombination between FRT containing transposons that flank the *cph*-coding sequence (see Materials and Methods). *cph* mutants were embryonic lethal, progressing to late stages of embryogenesis. The expression of early temporal factors Hb and Kr was normal; however, *cph* mutants displayed abnormal temporal expression patterns of Pdm and, in particular, Cas. Whereas Pdm expression is normally repressed at approximately stage 11, *cph* mutants displayed prolonged Pdm expression in a subset of NSCs at stages 11 and 12 (Fig. 3A-B′). More dramatically, *cph* mutants displayed a severe reduction in Cas expression during stages 11-13 (Fig. 3C-D″). Taken together, these phenotypes indicate a defect in the Pdm-to-Cas transition. Based on the relative severity of the phenotypes and previous analysis of the interaction between Pdm and Cas, we propose that the primary defect in *cph* embryos is the failure to induce Cas. Cas is known to repress Pdm expression and loss of Cas leads to extended Pdm expression (Kambadur et al., 1998; Grosskortenhaus et al., 2006). These findings are consistent with our observation that the timing of Cph expression in the neural stem cell correlates with the transition from Pdm to Cas. We conclude that *cph* mutants are defective in the transition from Pdm to Cas as a result of a failure in Cas induction.

Given that Cph acts at the Pdm-to-Cas transition, we investigated whether earlier temporal factors activate Cph expression. *pdm* mutants exhibit no changes in Cph::YFP expression (Fig. S1). This is consistent with the observation that the activation of *cas* is not delayed in *pdm* mutants. Neither loss of *kr* nor misexpression of Kr in NSCs (with *asense*-GAL4) affected the expression of Cph::YFP (Fig. S1). Therefore, the factors regulating Cph expression remain to be determined.

To investigate how disrupting the Pdm-to-Cas transition affects neuronal subtype specification, we examined the NB7-1 lineage in *cph* mutant embryos. As described above, the temporal cascade factors determine the birth order of the U neurons. Control embryos have exactly five U neurons per hemisegment, whereas *cph* embryos had 7.1±1.2 U neurons per hemisegment on average. Using a panel of markers to distinguish the U neuron subtypes, we determined that *cph* mutants contained extra U4 neurons, which normally are specified within the Pdm temporal window (Fig. S4A,B). Therefore, the defect in temporal identity that we observed in the NSCs correlates with the defect we observed in neuronal subtype specification. Specifically, the disrupted Pdm-to-Cas transition causes NB7-1 NSCs to generate excess Pdm-specified U4 neurons. We conclude that Cph acts as a switching factor and regulates the switch from U4 to U5 neuron production.

**Fig. 4.**
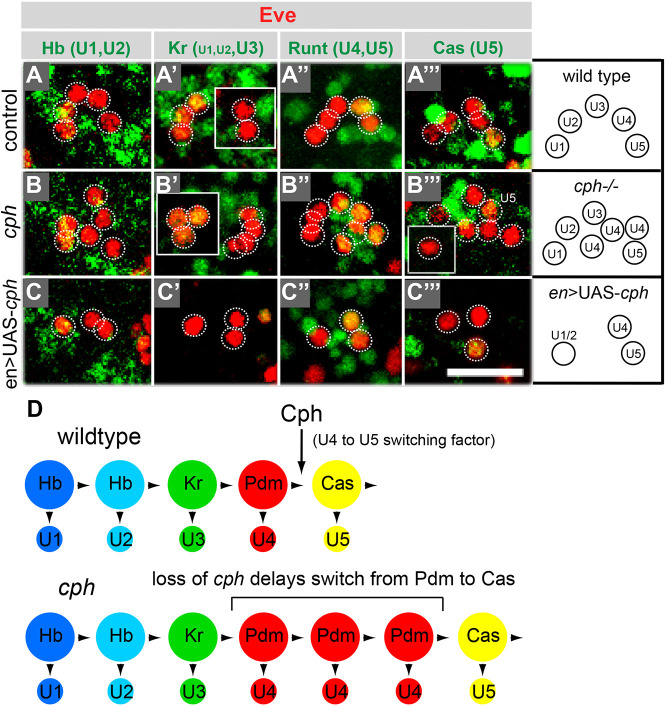
**Cph regulates neuron specification in the NB7-1 lineage.** (A-A‴) The NB7-1 lineage generates five U neurons. (B-C‴) *cph* mutants contain an excess of U4 neurons (B-B‴), and early misexpression of *cph* represses the generation of U1-U3 (C-C‴). U neurons are shown in individual hemisegments (maximum intensity projections). U neurons are Eve positive (red) and are outlined. Individual U neurons are identified by a combination of subtype markers (green): U1 and U2 express Hb; U1 and U2 weakly express Kr; U3 strongly expresses Kr; U4 and U5 express Runt; and U5 expresses Cas. All images are from stage 16 embryos. UAS-*cph* driven by *engrailed*-GAL4. Scale bar: 20 μm. *n*=6 embryos from three independent experiments. (D) Based on the *cph* expression pattern and the *cph* mutant phenotype, we propose that *cph* is a switching factor that promotes the transition between the ‘Pdm’ and ‘Cas’ temporal windows.

We next asked whether early misexpression of Cph in the neural stem cell is sufficient to induce expression of Cas and modify U neuron specification. Early misexpression of Cph using *engrailed*-GAL4 (which drives expression in a stripe of NSCs, including NB7-1) or *asense*-GAL4 (which drives expression in all NSCs) did not affect the timing of Cas expression (Fig. S1). Consistent with this, misexpression of Cph did not affect specification of U4 or U5 (Fig. 4C). Therefore, Cph is not sufficient to induce early Cas or promote the transition from specification of U4 to specification of U5. However, Cph misexpression did repress the generation of U1-U3 neurons, which are specified during the Hb/Kr temporal windows. On average only 2.9±1.2 U neurons were present in total, with 90% of hemisegments lacking U3, while U1 and U2 were alternatively repressed [typically only one or the other was present in a given hemisegment (U1/U2 identity distinguished based on Cph::YFP expression, see Fig. S2D] (Fig. 4C). Although the generation of these neurons was impaired, the neural stem cell expression of the corresponding temporal factors, Hb and Kr, was unaffected (Fig. S1). The loss of U1-U3 could be due to cell death, to reduced NSC proliferation or to a switch in cell fate. Co-expression of Cph with p35 did not rescue the loss of U1-U3 (Fig. S7), excluding cell death as the cause. Analysis of the mitotic index after Cph misexpression in NSCs, during the stages at which U1-U3 are generated, revealed a slight decrease but the difference was statistically insignificant (Fig. S8). Therefore, Cph appears to interfere with the ability of Hb and Kr to specify U1/2 and U3, respectively (see below).

A defining characteristic of temporal identity is that neuron specification is regulated at the level of the NSCs. Therefore, to determine whether Cph regulates U neuron fate within the neural stem cell or in neurons, we attempted to rescue the *cph* phenotype by preferentially expressing UAS*-cph* in either the NSCs or the post-mitotic neurons. Using *elav*-GAL4 to drive Cph in neurons, we were unable to rescue the extra U4 neuron phenotype in *cph* embryos (Fig. S2). In addition, neuron-specific expression did not disrupt U1-U3, as seen when Cph was misexpressed in the NSCs. In contrast, neural stem cell expression via *worniu*-GAL4 rescued the U neuron phenotype, resulting in five total U neurons and a single U4 neuron per hemisegment (Fig. S2). In this experiment, U1-U3 were unaffected, presumably because *worniu*-GAL4 did not drive expression early enough to repress their generation. When UAS-*cph* was driven with *asense*-GAL4 in a *cph* mutant, the loss of U1-U3 was equivalent to that observed when expressed in a control background (3.4±0.9 in *cph* mutant background versus 3.1±0.8 in control background). Overall, the observation that Cph acts within the stem cell rather than in neurons, supports our hypothesis that Cph regulates neural stem cell temporal identity. Specifically, our data indicate that Cph acts as a switching factor in the neural stem cell to promote the transition from the Pdm to Cas window, and thus regulate neuronal subtype specification (Fig. 4D).

### *cas* is a direct target of Cph

The NB7-1 lineage phenotypes of *cas* and *cph* mutants are nearly identical, suggesting that the primary defect of *cph* in this context is a failure to induce *cas* expression. Similar to *cph* mutants, *cas* mutants have prolonged Pdm expression and generate excess U4 neurons (Grosskortenhaus et al., 2006). Both U4 and U5 neurons express Runt, but only U5 expresses Cas (Pearson and Doe, 2003). Based on expression of a *cas-lacZ* reporter, *cas* mutants were shown to lack U5 (Grosskortenhaus et al., 2006). However, Cph provides an additional marker of U5 fate, and we observed that *cas* mutants contain U neurons that correspond to U5 fate based on co-expression of Runt and Cph::YFP (Fig. S3). This suggests that the principal U neuron phenotype in both *cas* and *cph* mutants is additional U4 neurons. However, as Cph::YFP cannot be assayed in *cph* mutants, we continued our analysis using Runt expression as a marker for U4/U5 fate. In this regard, these mutants were nearly equivalent: both contained approximately four Runt-positive U4/U5 neurons per hemisegment (4.3±1.4 in *cas* and 4.1±1.2 in *cph*), whereas wild-type embryos contained exactly two. Whereas the expressivity of the *cas* U neuron phenotype is segment specific (Grosskortenhaus et al., 2006), the U4 phenotype in *cph* is fairly consistent throughout the ventral nerve cord (Figs S3D and S4).

**Fig. 5.**
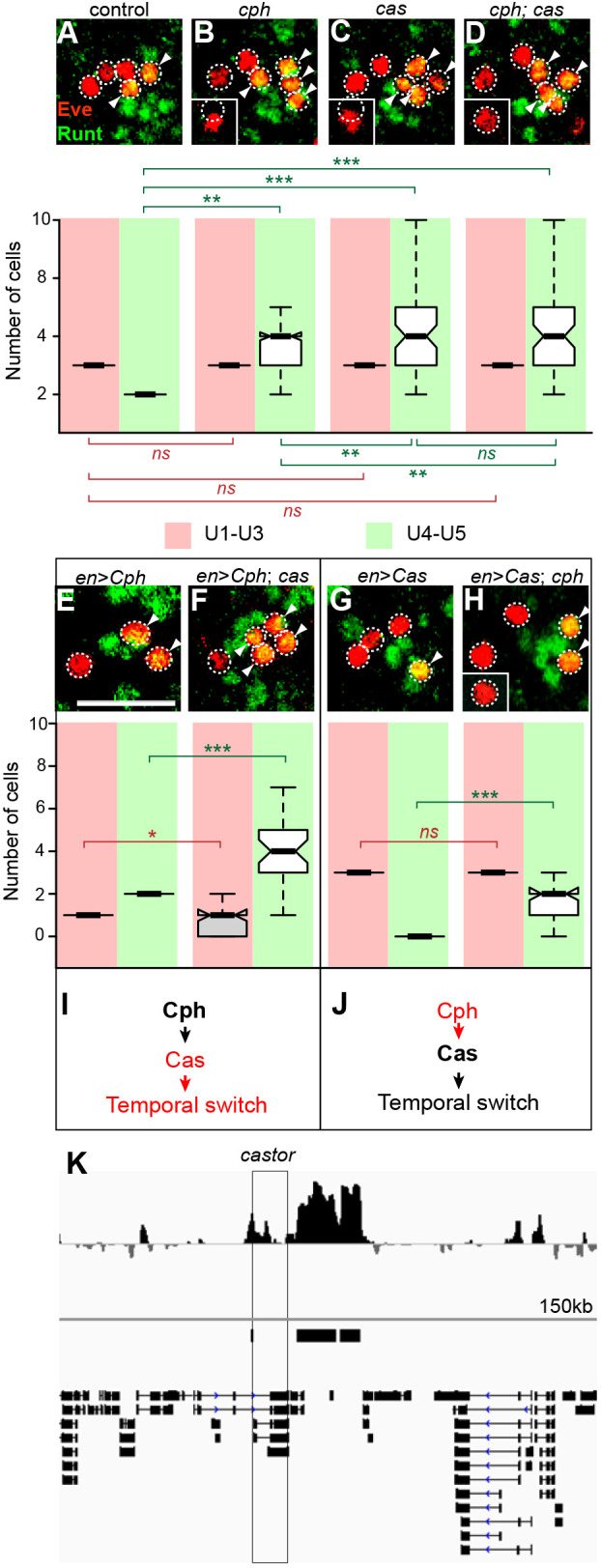
**Cph acts upstream of Castor in a simple linear pathway.** (A-D) Cph and Castor are in the same pathway. Wild-type embryos contain five U neurons and a single U4 and U5 neuron per hemisegment. (B-D) *cph* (B) and *cas* (C) embryos both contain excess U4/U5 neurons, and the phenotype of *cph;cas* double mutants (D) is equivalent to either single mutant. (E,F,I) Cph acts upstream of Cas. Transgene expression of Cph represses early U neuron generation (E) but does not rescue excess U4 and U5 neuron generation in *cas* mutants (F). Box and whiskers plot shows median values (middle bars) and first to third interquartile ranges (boxes); whiskers indicate maximum and minimum values. (G,H,J) Cas acts downstream of Cph. Transgene expression of *cas* represses U4 and U5 generation, and suppresses the extra U4 and U5 neuron phenotype in *cph* mutants (H). Each panel shows U neurons from an individual hemisegment (maximum intensity projection). All U neurons are Eve positive (red); U4 and U5 neurons co-express Runt (green). Arrowheads indicate Eve and Runt double-positive U4 and U5 neurons. Scale bar: 20 μm; *n*=6 embryos from three independent experiments. A Mann–Whitney test was used to test for statistical significance between the experimental condition and control (***P<0.001; **P<0.01; *P<0.05). The number of U1-U3 neurons (Eve^+^/Runt^−^, red), and U4 and U5 neurons (Eve^+^/Runt^+^, green) is indicated in the corresponding box and whisker plots. (K) *cph* binds to the Castor promoter region (Targeted DamID trace).

To investigate whether *cas* and *cph* act in a single pathway to limit U4 generation, we examined *cph; cas* double mutants. The double mutants contained a statistically indistinguishable number of Runt^+^ U4/5 neurons (4.7±1.3) when compared with either *cph* (4.1±1.2) or *cas* (4.3±1.4) single mutants (Fig. 5A-D), suggesting that *cph* and *cas* act in the same pathway. What is the relationship between *cph* and *cas* within this pathway? The observation that *cph* is required for Cas expression places *cph* upstream of *cas*. If so, *cas* should then be dispensable for *cph* expression. Therefore, we examined *cph* expression in *cas* embryos. Loss of *cas* (or early misexpression) did not affect Cph::YFP expression (Fig. S3A-B′).

We tested this hypothesis further by performing two genetic epistasis experiments. First, we tested whether the ability of *cph* to limit U4 generation depended on *cas*. Indeed, misexpression of Cph in a *cas* mutant failed to rescue the extra U4 neuron phenotype (Fig. 5E,F). This supports our hypothesis that *cph* acts upstream of, and depends on, *cas* to regulate U4 neuron generation (Fig. 5I). Second, we tested whether Cas is able to regulate U4 generation independently of *cph*. By driving Cas expression in a *cph* mutant, we found that Cas restored the generation of a single U4 neuron (Fig. 5G,H). This indicates that Cas can act independently of *cph* to regulate U neuron specification (Fig. 5J). Taken together, these results support a model in which *cph* and *cas* act in a simple, linear pathway that promotes the switch from the Pdm temporal window to the Cas temporal window.

Given that Cph is necessary for Cas expression, we hypothesised that Cph binds directly to *cas* and regulates its transcription. This was supported by our observation that *cph* mutants have reduced *cas* mRNA expression (Fig. S5). To identify the genome-wide binding targets of Cph, we performed Targeted DamID (TaDa) (Southall et al., 2013; Marshall and Brand, 2015; Marshall et al., 2016) by expressing Cph fused to *E.coli* Dam methylase (see Materials and Methods). TaDa revealed 1624 Cph target genes within 1 kb of a significant binding peak, including a peak upstream of *cas* within known regulatory sequences (Kuzin et al., 2012) (Fig. 5K). Assigning a score to each of these peaks, the peak upstream of *cas* ranked 20/1624 (Fig. S6). Taken together, our genetic and molecular evidence support a model in which Cph directly regulates Cas expression, thereby promoting the temporal identity transition from Pdm to Cas within NSCs.

### Cph represses specification of early temporal fate

The observation that early misexpression of Cph represses the generation of the early U1-U3 neurons suggested that *cph* also regulates other aspects of temporal identity. We further investigated the loss of U1-U3 by analysing reporter expression from a split-GAL4 NB7-1 lineage tracer (Kohwi et al., 2013). Reporter expression at stage 12 was consistent with the loss of U1-U3, and suggested that growth of the lineage was affected (Fig. S7). We conclude that precocious expression of Cph interferes with the generation of U1-U3 by altering their cell fate and/or reducing the proliferation of NB7-1. Importantly, these alterations in the lineage occur despite normal expression of Hb and Kr in the NSCs (Fig. S1).

Previous studies have shown that NSCs undergo changes in competence that limit their ability to generate specific neuronal subtypes. This was deduced by ectopically expressing temporal factors, such as Hb or Kr, and asking whether NSCs were able to induce the generation of the corresponding neurons. For example, in the NB7-1 lineage, ectopic expression of Hb or Kr is sufficient to specify the generation of U1/U2 or U3 neurons, respectively, but only until approximately the fifth neural stem cell division (Isshiki et al., 2001; Pearson and Doe, 2003; Cleary and Doe, 2006; Kohwi et al., 2013). The failure to generate these neurons after this stage reveals that the neural stem cell has lost competence to do so.

**Fig. 6.**
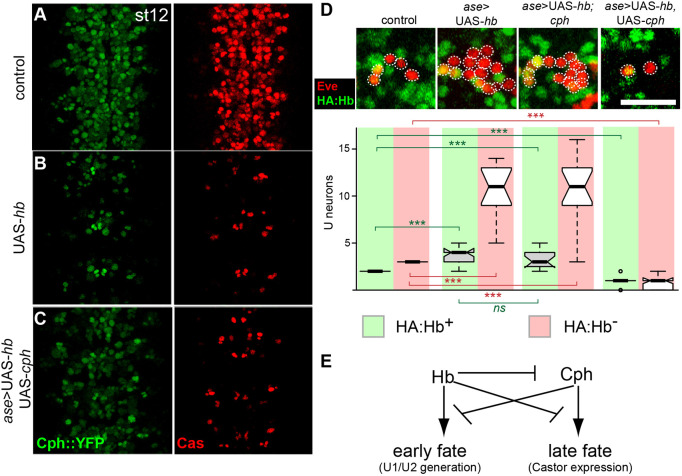
**Cph and Hb have cross-inhibitory activities.** (A,B) Misexpression of Hb represses Cph expression (from the endogenous locus) and Cas. (C) Co-expression of a Cph transgene suppresses the repressive effect Hb exerts on the endogenous Cph locus. The repression of Cas by Hb is independent of Cph. Pan-neural stem cell expression of *hb* and *cph* was driven by *asense*-GAL4. Panels show ventral views of the NSC layer of stage 12 embryos; Cph::YFP protein trap expression from the endogenous *cph* locus (green) and Castor expression (red). *n*=12 embryos from three independent experiments. (D) Prolonged misexpression of Hb also generates extra U1 and U2 neurons, and a loss of *cph* does not extend NSC competence to Hb. However, misexpression of Cph is sufficient to repress the effects of Hb misexpression on neuronal subtype production, suggesting that Cph is sufficient to end the Hb competence in NSCs. Panels show U neurons (Eve positive, red) from individual hemisegments (maximum intensity projection) at stage 16. U1 and U2 neurons co-express HA tagged Hb reporter (green). Pan-neural stem cell expression of *hb* and *cph* was driven by *asense*-GAL4. Scale bar: 20 μm; The number of U neurons in the control and experimental conditions is indicated in the corresponding box and whisker plots [median values (middle bars) and first to third interquartile ranges (boxes); whiskers indicate maximum and minimum values]. *n*=6 embryos from three independent experiments. A Mann–Whitney test was used to test for statistical significance (***P<0.001). (E) Summary and model of the cross-inhibitory interactions between Cph and Hb.

In wild-type embryos, Cph expression begins at approximately the same time that competence to generate U1-U3 is lost. Misexpression of Hb strongly repressed Cph in the NSCs (Fig. 6A-C) and consistent with this result, *cph* was not required for ending the competence window (Fig. 6D). Further investigation of Cph and Hb revealed complex interactions with regards to neural stem cell expression and neuron specification, suggesting that these genes have cross repressive activities (Fig. 6E).

**Fig. 7.**
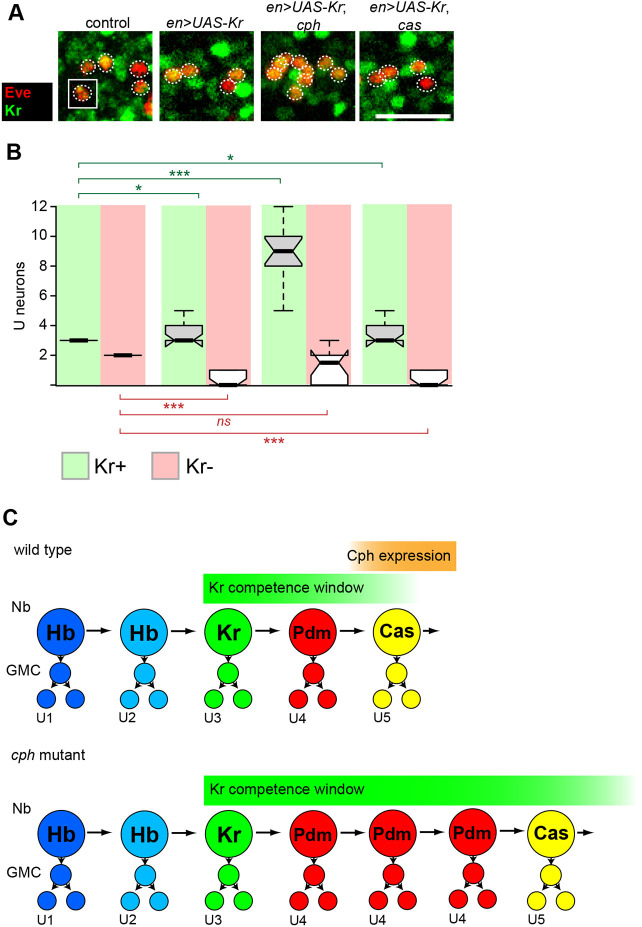
**Cph limits neural stem cell competence to respond to Kr.** (A,B) Prolonged misexpression of Kr limits competence to generate U3 neurons. Loss of *cph* extends this competence window and allows the generation of a large number of extra U3 neurons due to prolonged Kr misexpression. This phenotype is not observed in *cas* mutants, indicating that Cph regulates the Kr competence window independently of Cas. Images show U neurons (Eve positive, red) from individual hemisegments (maximum projections) at stage 16. Co-expression of Kr was used to identify U3 neurons. Hemisegments normally contain three Kr-positive neurons: U1, U2 and U3. As Kr does not affect the presence of U1 and U2, as confirmed by Hb expression (Cleary and Doe, 2006), we assume that in all experiments two of the Kr-positive neurons are U1 and U2. Scale bar: 20 μm. The number of U neurons in the control and experimental conditions is indicated in the corresponding box and whisker plots [median values (middle bars) and first to third interquartile ranges (boxes); whiskers indicate maximum and minimum values]. *n*=6 embryos from three independent experiments. A Mann–Whitney test was used to test for statistical significance (***P<0.001; *P<0.05). (C) Model of Cph function in the NB7-1 lineage. Progression of the temporal cascade and regulation of independent temporal competence windows in the NB7-1 lineage. Cph expression coincides with the transition from Pdm to Cas and the end of the Kr competence window. In *cph* mutants, the Pdm window and the Kr competence window are extended.

In contrast to Hb, prolonged Kr misexpression has no effect on Cph expression (Fig. S1B-B′). We therefore analysed whether *cph* regulates competence to generate Kr-specified neurons. Prolonged expression of Kr leads to the generation of one or two extra U3 neurons in the NB7-1 lineage (Fig. 7A) (Cleary and Doe, 2006; Touma et al., 2012). We therefore misexpressed Kr in a *cph* mutant and asked whether additional U3 neurons were generated. Whereas misexpression of Kr in a wild-type background generated 1.4±1.0 U3 neurons, misexpression of Kr in a *cph* mutant generated 6.8±1.0 U3 neurons (mean±s.e.m.) (Fig. 7A). This is consistent with our results above, where early misexpression of Cph repressed U3 neuron generation (Fig. 4C). We conclude that Cph is both necessary and sufficient to limit the competence of NSCs to generate U3 neurons.

Our analysis of the role of Cph as a switching factor indicated that *cph* acts upstream of *cas* to promote the transition from the Pdm to Cas temporal window. Therefore, we wondered whether the same pathway regulates neural stem cell competence to generate U3 neurons. However, in contrast to *cph*, misexpression of Kr in a *cas* mutant did not extend the ability of Kr to specify U3 fate (Fig. 7A,B). This suggests that the Cph>Cas pathway that regulates the switch from U4 to U5 generation does not regulate neural stem cell competence. In addition, we note that in a previous experiment, early misexpression of Cph in a *cas* mutant was sufficient to repress U1-U3 generation (Fig. 5F). Taken together, our data suggest that in the NB7-1 lineage Cph has two genetically separable activities: (1) Cas-dependent switching factor activity (which affects the U4 to U5 switch); and (2) Cas-independent repression of the Kr competence window (which affects U3 generation).

## DISCUSSION

Our results establish Cph as a switching factor that regulates the transition from Pdm to Cas (Fig. 7C). In addition, we find that Cph can affect earlier temporal identity programs by interfering with the activity of Hb and Kr. We conclude that Cph regulates the competence of NSCs to respond to these factors. Therefore, Cph provides a molecular link between the progression of temporal identity and the loss of neural stem cell competence.

### Cph is a temporal cascade switching factor

The role of the temporal cascade, Hb, Kr, Pdm and Cas, in regulating neuron subtype specification has been established. An important remaining question concerns how progression of the cascade is regulated (Kohwi and Doe, 2013). One facet of temporal cascade progression is the regulatory interactions among the temporal factors themselves. Based on misexpression experiments, each factor seems to induce the expression of the next factor (Isshiki et al., 2001). In addition to this, repressive interactions occur whereby temporal factors repress expression of the preceding factor as well as the factor after the next one. However, loss-of-function experiments reveal that these interactions are unnecessary for the overall timing of each temporal window; the progression between temporal windows appears to require additional regulatory mechanisms that involve switching factors.

All previously reported switching factors function by repressing the initial temporal factor in the sequence. Cph, in contrast, is the first example of a switching factor in the VNC that promotes the switch between sequential temporal factors by inducing the second factor rather than repressing the initial factor: Cph is crucial for induction of Cas but not for repression of Pdm.

The ultimate consequence of temporal regulation of NSCs is the generation of distinct neuronal subtypes over time. In the NB7-1 lineage, the transition from Pdm to Cas corresponds to a switch in generating U4 versus U5 motor neurons. We show that, in *cph* mutants, the defective switch from Pdm to Cas leads to the generation of excess U4 neurons. In this study, we focused on the effects of Cph on Eve^+^ motor neurons generated by the NB7-1 lineage. However, not all neurons of this lineage are marked by Eve and lineage analysis has identified a Nkx6^+^ (Eve^−^) VO motor neuron generated during the Kr^+^ Pdm^+^ window (Seroka et al., 2020). Although we have not investigated the effects of Cph on this neuron, it is intriguing that there are cells in close proximity to the U1-U5 neurons that express Cph::YFP and are Eve^−^ (Fig. 2D). Further work will be needed to elucidate whether the Nkx6^+^ neuron also expresses Cph and whether Cph has an effect on its specification, either via the NSC temporal cascade or another neuron-specific function.

### Cph regulates competence to respond to Hb and Kr

Similar to neural progenitors in the cerebral cortex, embryonic NSCs of the VNC eventually lose competence to specify early born neuron subtypes. Previous studies have shown that NB7-1 remains competent to generate Hb-specified U1/U2 neurons and Kr-specified U3 neurons until the fifth stem cell division (Pearson and Doe, 2003; Cleary and Doe, 2006; Kohwi and Doe, 2013). At this stage, misexpression of Hb or Kr fails to specify these neuron subtypes in the progeny. Our work demonstrates that Cph plays a role in repressing neural stem cell competence to generate U3 neurons. Cph also represses U1 and U2; however, it is not necessary for closing the Hb competence window.

### Conservation of temporal identity and neural stem cell competence

The study of neural stem cell competence in *Drosophila* has been an active area of research because it parallels the loss of competence that occurs in the precursor cells during development of the cerebral cortex and retina. Ikaros, a vertebrate homologue of Hb, also regulates the generation of early born neurons, and its activity is similarly limited to a competence window (Alsio et al., 2013). Another factor that exhibits a potentially conserved role in temporal identity is COUP-TF, a vertebrate homologue of Svp (Lim et al., 2018). Similar to Svp, which regulates the switch between the Hb and Kr temporal windows (Kanai et al., 2005; Mettler et al., 2006), COUP-TF regulates a switch in temporal identity between neurogenesis and gliogenesis (Naka et al., 2008; Tomassy et al., 2010).

In this study, we demonstrate that Cph and its vertebrate homolog, CTIP2, provide an additional similarity between the mammalian cerebral cortex and the *Drosophila* VNC. In the cerebral cortex, CTIP2 is expressed in deep layer neurons and regulates the fate of neurons that project to subcortical targets (Arlotta et al., 2005; Leyva-Díaz and López-Bendito, 2013). Interestingly, CTIP2, along with FEZF2, forms a pathway that regulates the identity of these relatively early born neurons that maintain CTIP2 expression (Chen et al., 2008). Within this pathway, FEZF2 expression within the precursor cells gives rise to post-mitotic expression of CTIP2. Therefore, although CTIP2 does not appear to regulate temporal identity directly within the precursor cells, its expression pattern depends on upstream temporal cues. We speculate that the regulation of precursor cell temporal identity by Cph may represent an ancestral function that, in the mammalian cerebral cortex, was subdivided by the FEZF2/CTIP2 pathway.

## MATERIALS AND METHODS

### Fly stocks

The Cph::YFP protein trap line CPTI-001740 contains an insertion (Morin et al., 2001) in the first intron of the *CG9650* RL isoform and is similarly included in all other annotated *CG9650* isoforms, except the smallest RJ isoform. The insertion is homozygous viable and overtly wild type. The *cph^1^* deletion was generated by recombination of FRT insertion elements flanking the entire RL isoform transcript using the piggyBac transposon insertion lines d03295 (which is inserted upstream of *cph* into position X:7,195,692) and f06617 (which is inserted downstream of *cph* into position X:7,241,777) (Parks et al., 2004; Thibault et al., 2004). Recombination between these FRT sites removes 46 kb of genomic sequence, within which *cph* is the only annotated gene (FlyBase release 6). *cph^1^* mutants are embryonic lethal and arrest at a late stage of embryogenesis. The UAS-*cph* transgene was generated by integration of the pUAST-attB-Cph construct into *attP2*. pUAST-attB-Cph was generated by amplification of the RL isoform from a cDNA library using the primers: GAGAGAATTCATGTACAACAAAATGCTGTCGATC and GAGATCTAGATCATGCCTCCTCCTTAAGCGA, and then inserted between the EcoRI and XbaI cloning sites of pUASTattB (Bischof et al., 2007). The NB7-1 lineage phenotype of *cph^1^* mutants could be rescued by *worniu*-GAL4 driven expression of UAS-*cph* (Fig. S9). The Cph targeted DamID transgene was generated by integration of the pUASTattB-LT3-NDam-Cph construct into *attP2*. The RL isoform of *cph* was amplified using the primers CAGAAACTCATCTCTGAAGAGGATCTGCGAATGTACAACAAAATGCTGTCG and AGAGGTACCCTCGAGCCGCGGCCGCAGATCTCATGCCTCCTCCTTAAGC, and inserted into the BglII cloning site of pUASTattB-LT3-NDam (Southall et al., 2013) by Gibson assembly (Gibson et al., 2009). The following additional mutants and alleles were used: *hb^P^hb^FB^* (Hulskamp et al., 1994), *kr^1^kr^CD^* (Romani et al., 1996), Df(2L)ED733 (Grosskortenhaus et al., 2006), *cas^24^* (Cui and Doe, 1992), UAS-*cas* (Kambadur et al., 1998), UAS-*hb* (Bloomington #8503), UAS-*kr* (Hoch and Jackle, 1998), UAS-*HA:pdm2* (Grosskortenhaus et al., 2006), *HA:hb* (Kohwi et al., 2013), UAS-p35 (Bloomington 5073), *asense*-GAL4 (a gift from Andrew Jarman, University of Edinburgh, UK), *engrailed*-GAL4 (Bloomington 30564), *worniu*-GAL4 (Albertson et al., 2004) and *elav*-GAL4 (Bloomington 5146). Mutations were balanced over *FM7c Dfd-YFP*, *CyO Hb-lacZ*, *CyO Kr-GFP*, *TM3 ftz-lacZ* or *TM3 twi>GFP*.

### Immunofluorescence, fluorescence *in situ* hybridization and confocal microscopy

Embryos were staged, collected and fixed according to standard methods. The following antibodies, with dilutions and sources, were used: mouse anti-Eve 3C10 [1:100, Developmental Studies Hybridoma Bank (DSHB)], mouse anti-Engrailed 4D9 (1:50, DSHB), rat anti-Elav 7E8A10 (1:30, DSHB), guinea pig anti-Krüppel (1:500, Asian Distribution Center for Segmentation Antibodies), rabbit anti-Castor (1:25, provided by Chris Doe, University of Oregon, OR, USA), rabbit anti-Hunchback (1:500, Asian Distribution Center for Segmentation Antibodies), rabbit anti-Runt (1:500, Asian Distribution Center for Segmentation Antibodies), mouse anti-Pdm (Nubbin) (1:100, provided by Steve Cohen, University of Copenhagen, Denmark), rat anti-HA (1:20, Roche, 11867423001), anti-digoxigenin-POD, Fab fragments (1:500, Roche, 11633716001), chicken anti-β-Galactosidase (1:1000, Abcam, ab9361), rat anti-phospho-histone-H3 (1:500, Abcam, ab10543), chicken anti-GFP (1:1000, Abcam, ab13970) and guinea pig anti-Deadpan (1:5000; Caygill and Brand, 2017). The following secondary antibodies were used (1:200 dilution): Alexa Fluor goat anti-mouse 405 (A-31553), Alexa Fluor goat anti-chicken 488 (A-11039), Alexa Fluor goat anti-mouse (A-11004), anti-guinea pig (A-11075), anti-rabbit (A-11011) and anti-rat (A-11077) 568 and Alexa Fluor goat anti-mouse (A-21136), anti-rabbit (A-21070) and anti-rat (A-21094) 633 (Invitrogen). The *castor* antisense RNA probe was transcribed from a PCR template amplified with the following primers: GCCAGAGTTTAAGGAGTAGG and CAGTAATACGACTCACTATTAGCCTGCACTCGCTCTATCAA. Fluorescence *in situ* hybridization was performed using a TSA Plus Fluorescein kit (PerkinElmer). Embryos were imaged using a Leica SP2 or SP8 confocal microscope. For comparison of protein levels, samples were incubated with the same antibody dilution and imaged/processed with identical settings.

### Software

Confocal stacks were analysed in ImageJ and images for publication were arranged using Adobe Photoshop and Adobe Illustrator. Statistical analysis was performed and graphs were produced with Prism Graphpad.

## Supplementary Material

Supplementary information
