# Supplementary material for: The Drosophila homologue of CTIP1 (Bcl11a) and CTIP2 (Bcl11b) regulates neural stem cell temporal patterning
Source: Development. 2022 Sep 7;149(17):dev200677. doi: 10.1242/dev.200677 (PMC9482335; doi:10.1242/dev.200677)
Supplement: Supplementary information [file develop-149-200677-s1.pdf]

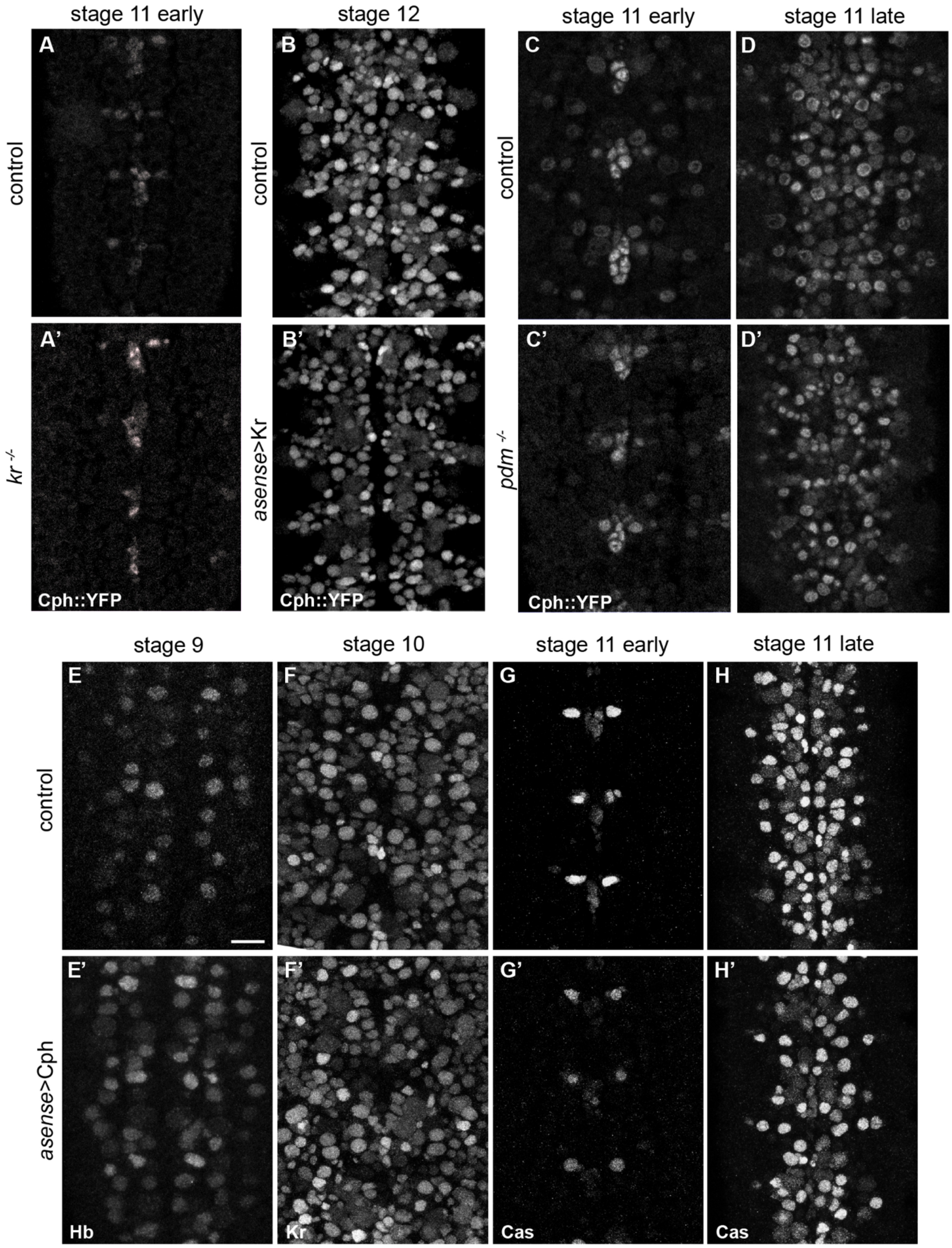


Figure 1

Development • Supplementary information

**Fig. S1. Early misexpression of Cph does not interfere with temporal cascade progression.**

(A-B') Neither loss of Kr function (*kr$^{1}$kr$^{CD}$*) nor misexpression of Kr impacts the timing of Cph. (C-D') Loss of *pdm* function (Df(2L)*Pdm*) does not impact Cph expression. Ventral view of the VNC of wild type embryos and embryos misexpressing *cph* (*asense*-GAL4 driving UAS-*cph*) shows no change to (E-E') Hb, (F-F') Kr and (G-H') Cas timing. Scale bars represent 20μm. For each condition, n=6 embryos were observed from 3 independent experiments.

Development • Supplementary information

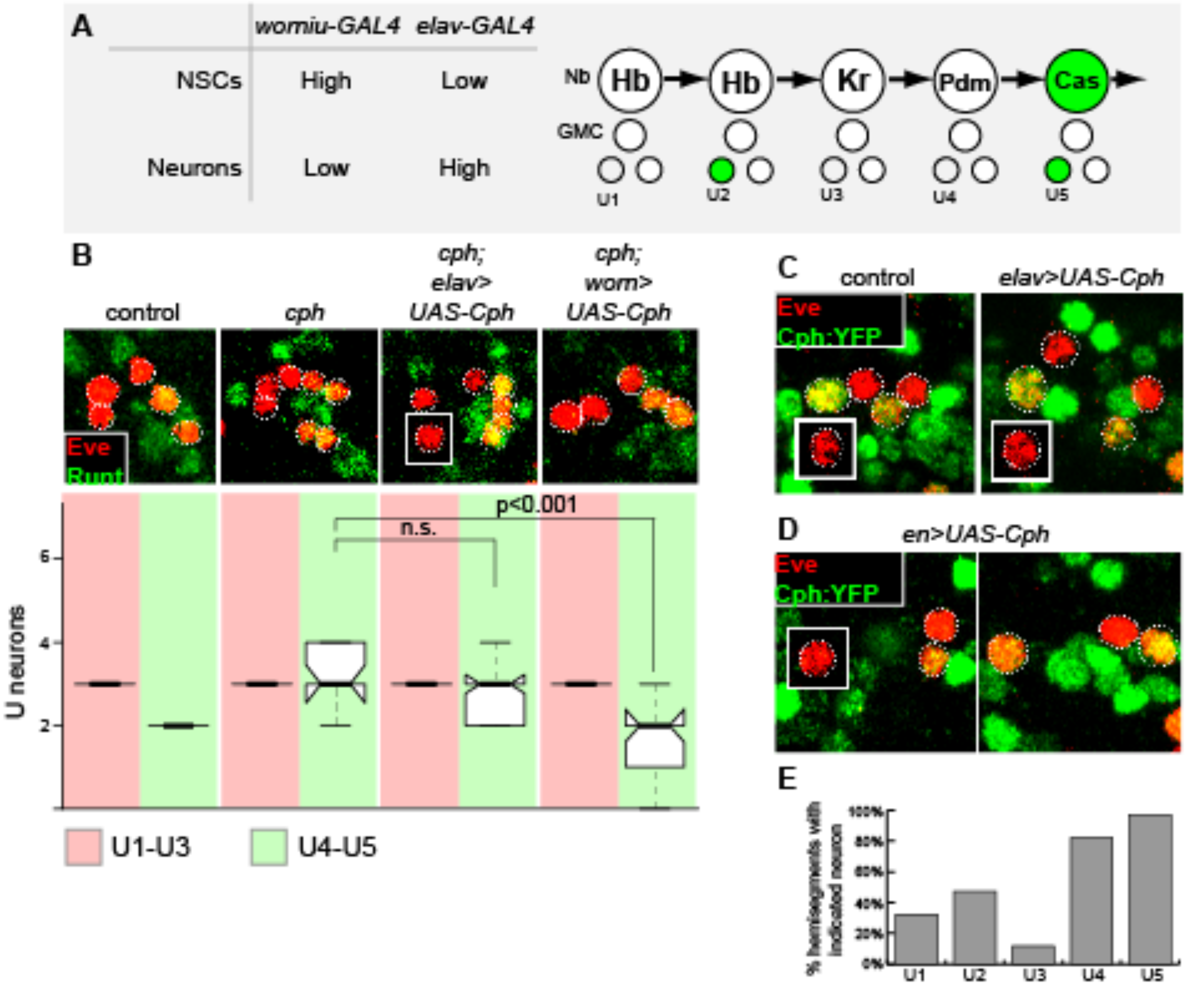


Figure 2

**Fig. S2. Cph acts in neural stem cells to regulate temporal identity.**

(A) Model of the first five divisions of the NB7-1 lineage. *worniu*-GAL4 and *elav*-GAL4 drive expression preferentially in the neural stem cell and in neurons, respectively. (B) Rescue analysis of *cph* U neuron phenotype. UAS-*cph* was driven in *cph* mutants using *worniu*-Gal4 or *elav*-Gal4. Whereas *worniu*-GAL4 rescued the U neuron phenotype nearly completely, *elav*-GAL4 had no effect. Error bars represent standard deviation.  n=6 embryos from three independent experiments. (C) Analysis of U neurons misexpressing Cph . Cph::YFP is expressed from the endogenous promotor and is present in U2 and U5. Misexpression of Cph in neurons using *elav*-GAL4 does not alter the pattern of Cph::YFP expression. (D)

Development • Supplementary information

Misexpression of Cph using *engrailed*-GAL4 interferes with generation of U1, U2 and U3. Although *cph* is normally expressed in U2, misexpression of UAS-*cph* in the neural stem cell interferes with U2 generation. Loss of U1 versus U2 was not favoured in this misexpression experiment. (E) The percentage of hemisegments that have U1-U5 neurons. n=5 embryos.

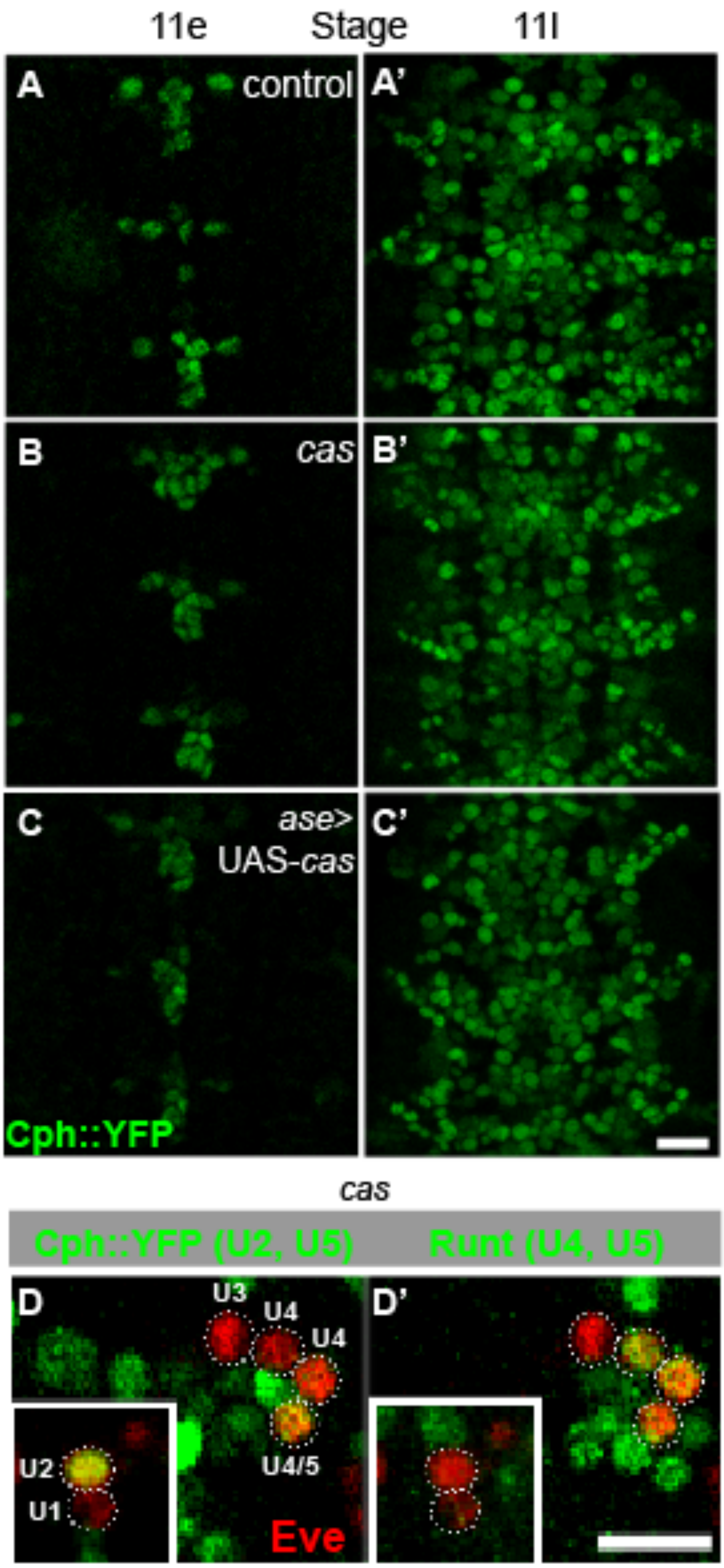


Figure 3

Development • Supplementary information

**Fig. S3. Castor does not regulate *cph* expression.**

(A-C') Ventral view of neural stem cell layer of embryos from early or late stage 11, stained for Cph::YFP expression (green). Ectopic Castor expression (B) was driven with *asense*-GAL4. (D-D') Maximum intensity projection of Eve positive (red) U neurons from individual hemisegments from stage 16 $cas^{24}$ mutant embryos. Expression of Cph::YFP (D) and Runt (D') from the same hemisegment was used to identify individual U neuron subtypes. $cas^{24}$ mutant hemisegments contain excess U4 neurons, but also contain a Cph and Runt positive U neuron that at least partially corresponds to U5 fate. Scale bar represents 20µm for A-C' and 10µm for D-D'. For each condition, n=6 embryos were observed from three independent experiments.

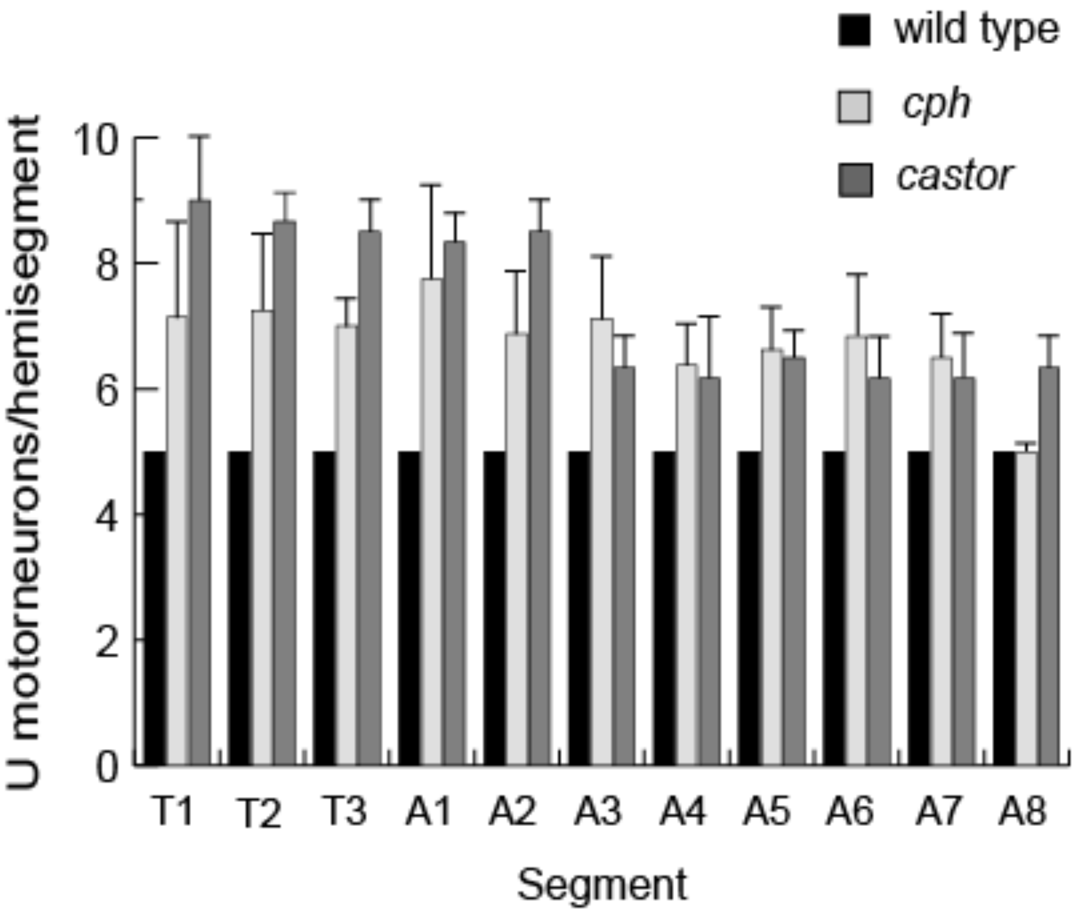


Figure 4

**Fig. S4. The *cph* U neuron phenotype is not segment specific.**

U neurons per hemisegment were scored based on segment identity. *cas*$^{24}$ mutants display an excess U neuron phenotype that is more severe in abdominal segments. However, in *cph* mutants, the excess neuron phenotype did not depend on segment identity. Error bars show standard deviation, n=5 embryos per genotype from 3 independent experiments.

Development • Supplementary information

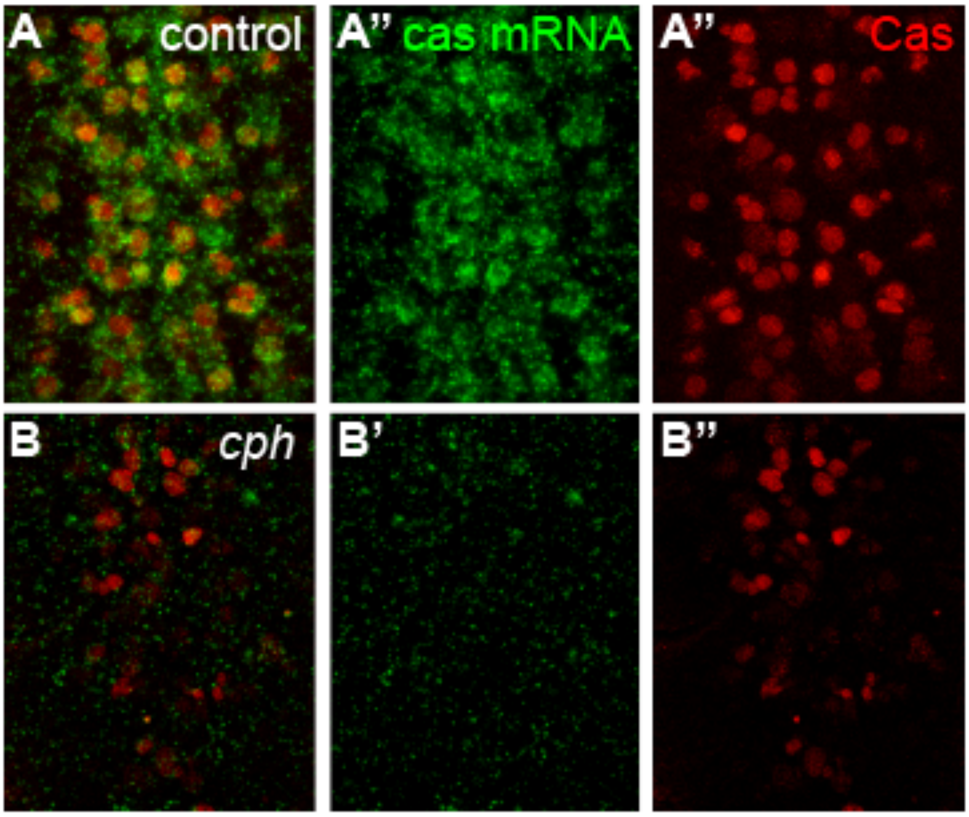


Figure 5

**Fig. S5.** Cph **regulates Cas transcription.**

Wild type (A) or *cph* (B) stage 12 embryos. *in situ* hybridisation against *castor* mRNA (green) and Castor antibody staining (red). Ventral view of the neural stem cell layer.

Development • Supplementary information

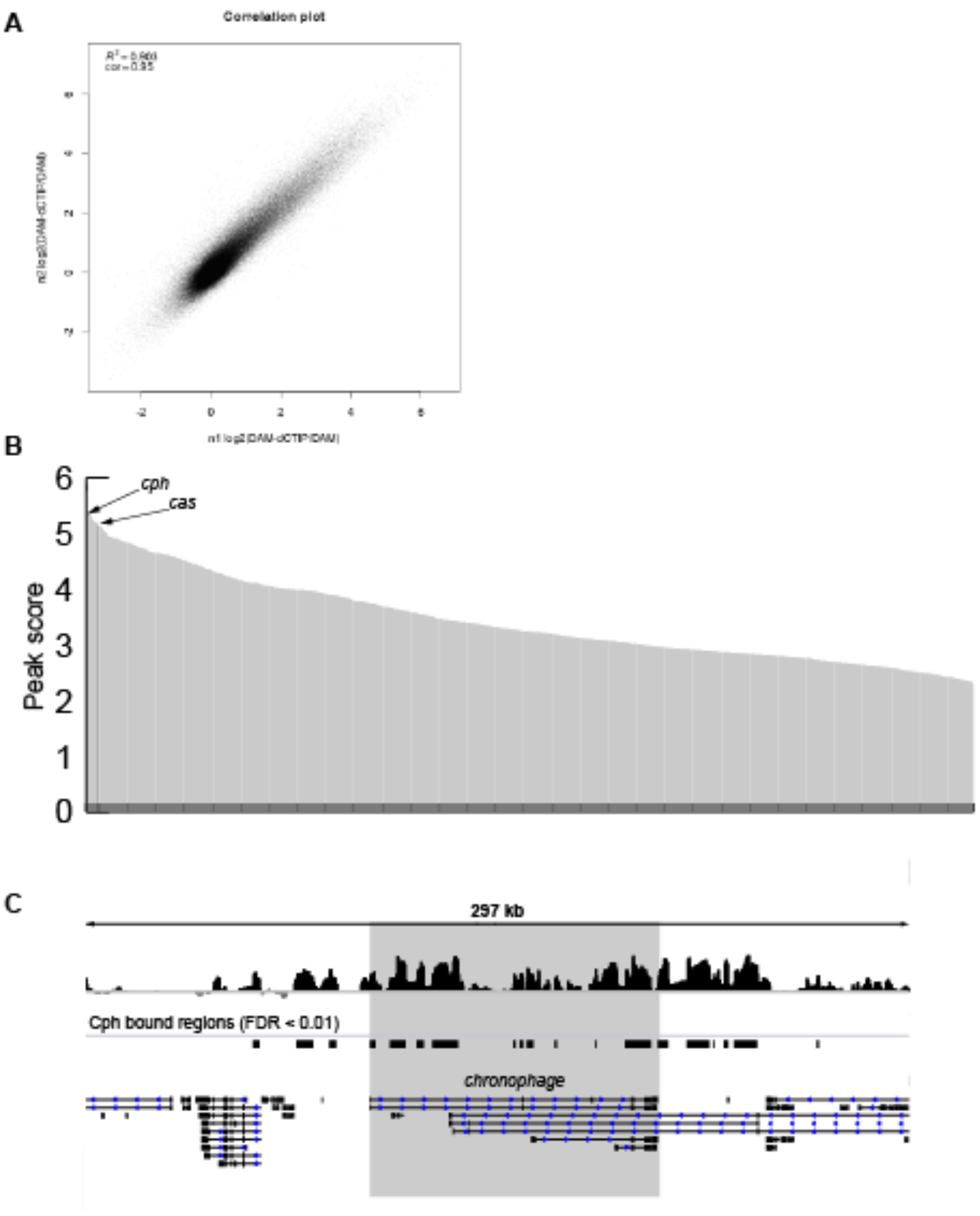


Figure 6

**Fig. S6. Targeted DamID to detect Cph genomic binding sites.**

(A) Correlation plot of two biological replicates of Cph Targeted DamID experiments.

(B) Rank order chart of all 1624 target genes bound by Cph. The *cph* peak score ranked 2/1624 and *cas* ranked 20/1624. (C) Binding profile of the *cph* locus.

Development • Supplementary information

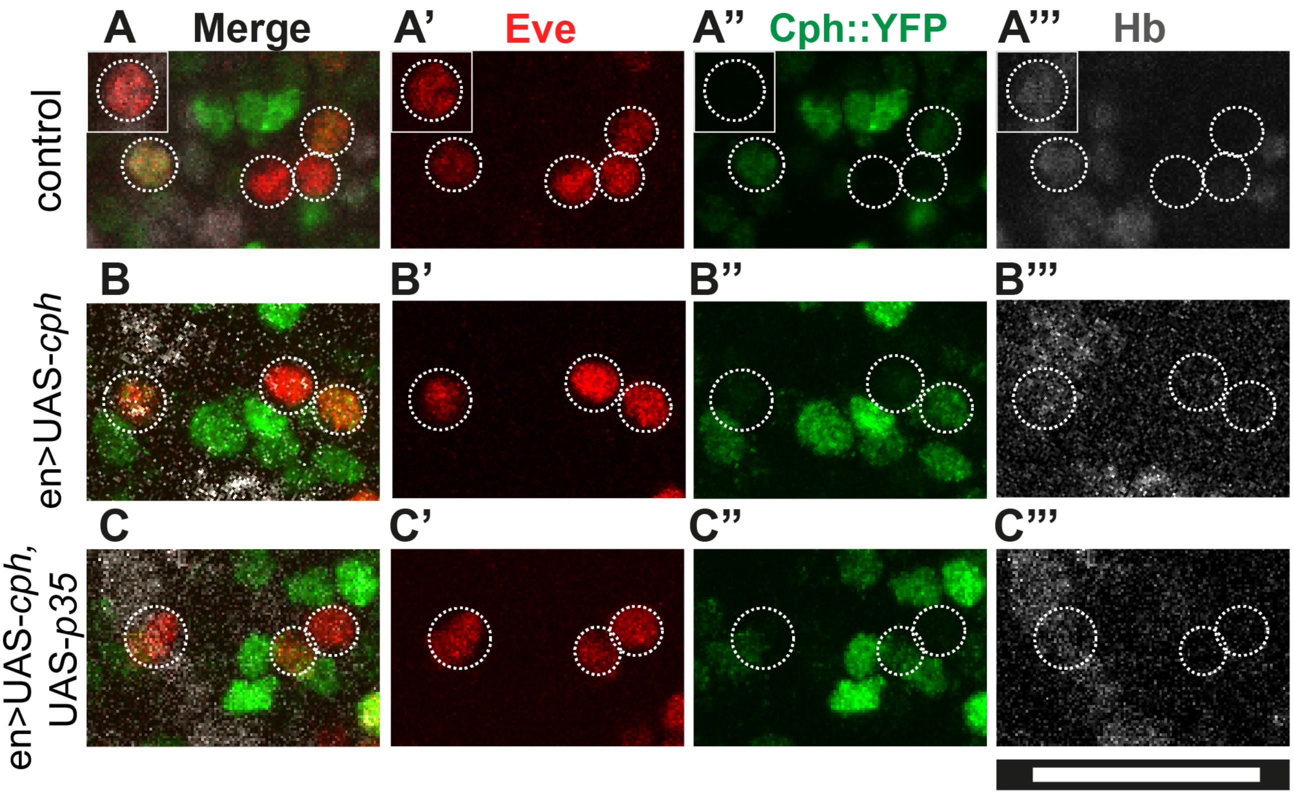


**Fig. S7. Preventing apoptosis does not rescue the loss of U1-U3 neurons upon Cph misexpression.**

(A-A'") Control embryos have U1-U5 neurons, of which U1-2 are Hb-positive. U2 and U5 express Cph::YFP. (B-B'") Misexpression of Cph (using *engrailed*-GAL4) results in a loss of U1-U3 neurons (U1/U2 are alternately repressed). (C-C'") Co-expression of UAS-Cph and UAS-p35 (with *engrailed*-GAL4) does not rescue the loss of U1-U3 neurons. Scale bars represent 20μm. n=5 embryos per genotype from 3 independent experiments.

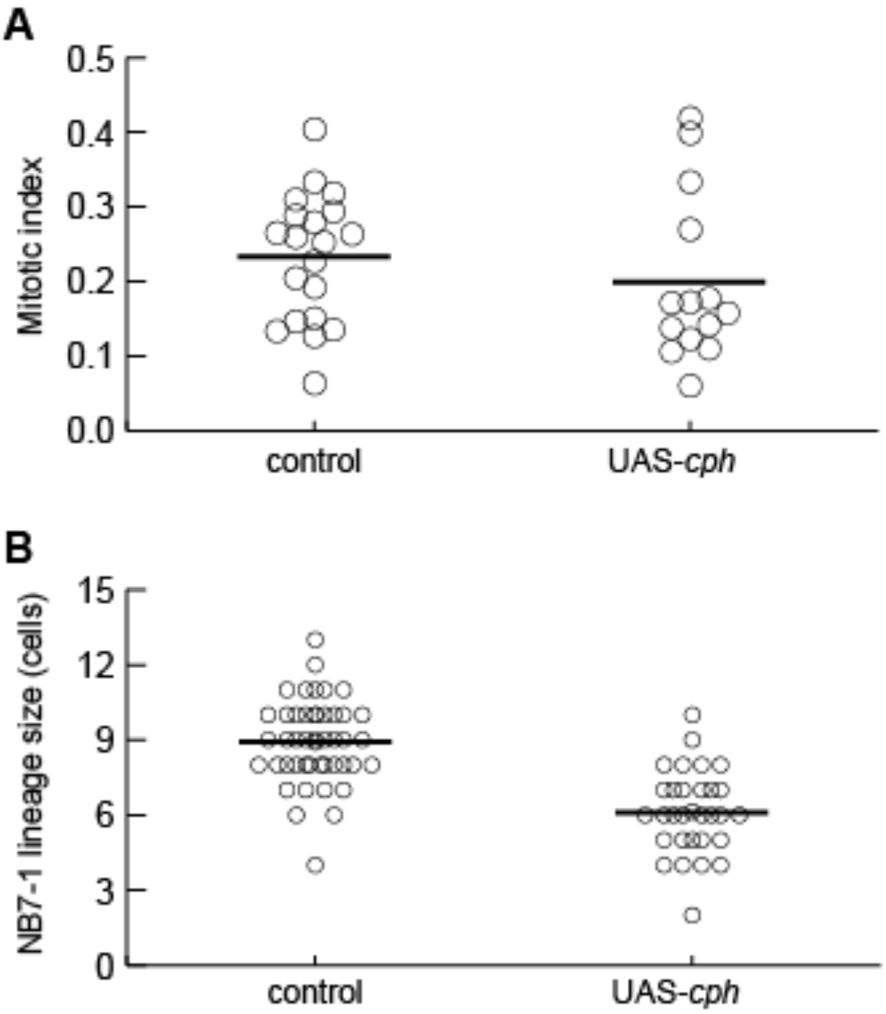


Figure 7

**Fig. S8. Misexpression of Cph has variable affects on the NB7-1 lineage.** (A) Mitotic index was scored in stage 9 and 10 NSCs, identified by Dpn and phospho-Histone H3 staining. Each data point represents an individual embryo obtained from 3 independent experiments. Approximately 100 NSCs were scored per embryo. No significant difference was observed between control embryos and *cph* misexpression embryos (*asense*-GAL4 was used to drive UAS-*cph*). (B) NB7-1 lineage size at stage 12 was scored using a NB7-1 lineage tracer (NB7-1 split GAL4). Lineage size after stage 12 was not scored as the expression of the lineage tracer became inconsistent. ($p<0.001$; Welch's t-test)

Development • Supplementary information

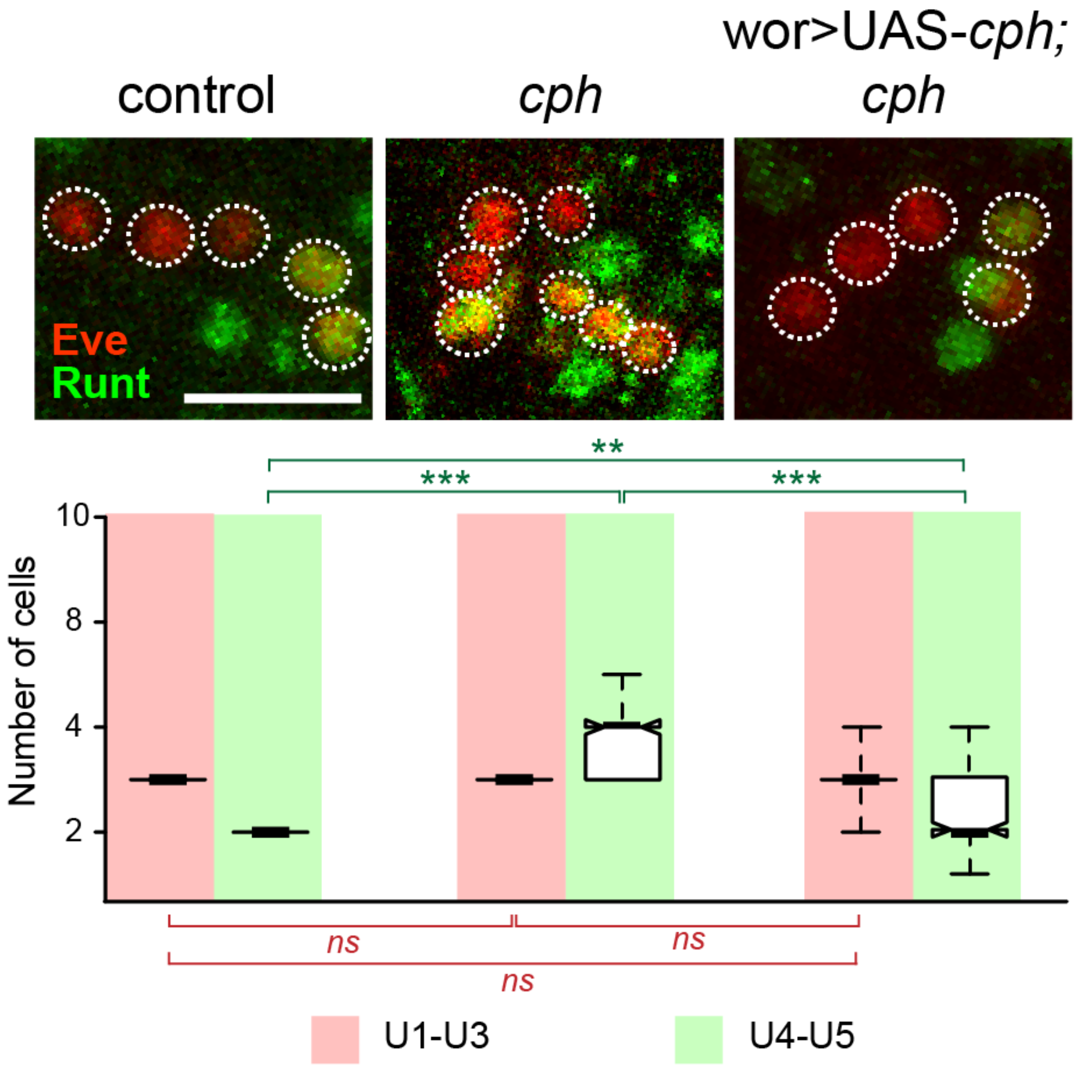


**Fig. S9. UAS-Cph rescues the *cph* mutant.**

Using *wor*-GAL4 to drive expression of UAS-Cph in a *cph* mutant rescues the number of U4-U5 neurons in the NB 7-1 lineage. In *cph* mutants there is an excess number of U4-U5 neurons but this number is reduced when Cph is expressed in NSCs. Scale bar represents 20µm; n= 6 embryos from 3 independent experiments. Mann-Whitney Test was used to test for statistical significance between the experimental condition and control (***, p<0.001; **, p<0.01). Plots represent median values (middle bars) and first to third interquartile range (boxes); whiskers indicate maximum and minimum values.

Development • Supplementary information
